# Supplementary material for: Distinct prefrontal top-down circuits differentially modulate sensorimotor behavior
Source: Nat Commun. 2020 Nov 26;11:6007. doi: 10.1038/s41467-020-19772-z (PMC7691329; doi:10.1038/s41467-020-19772-z)
Supplement: Supplementary file 1 — Supplementary Information [file 41467_2020_19772_MOESM1_ESM.pdf]

Supplementary Information

Distinct prefrontal top-down circuits differentially modulate sensorimotor behavior

Huda et al.

8 Supplementary Figures

Supplementary Figures

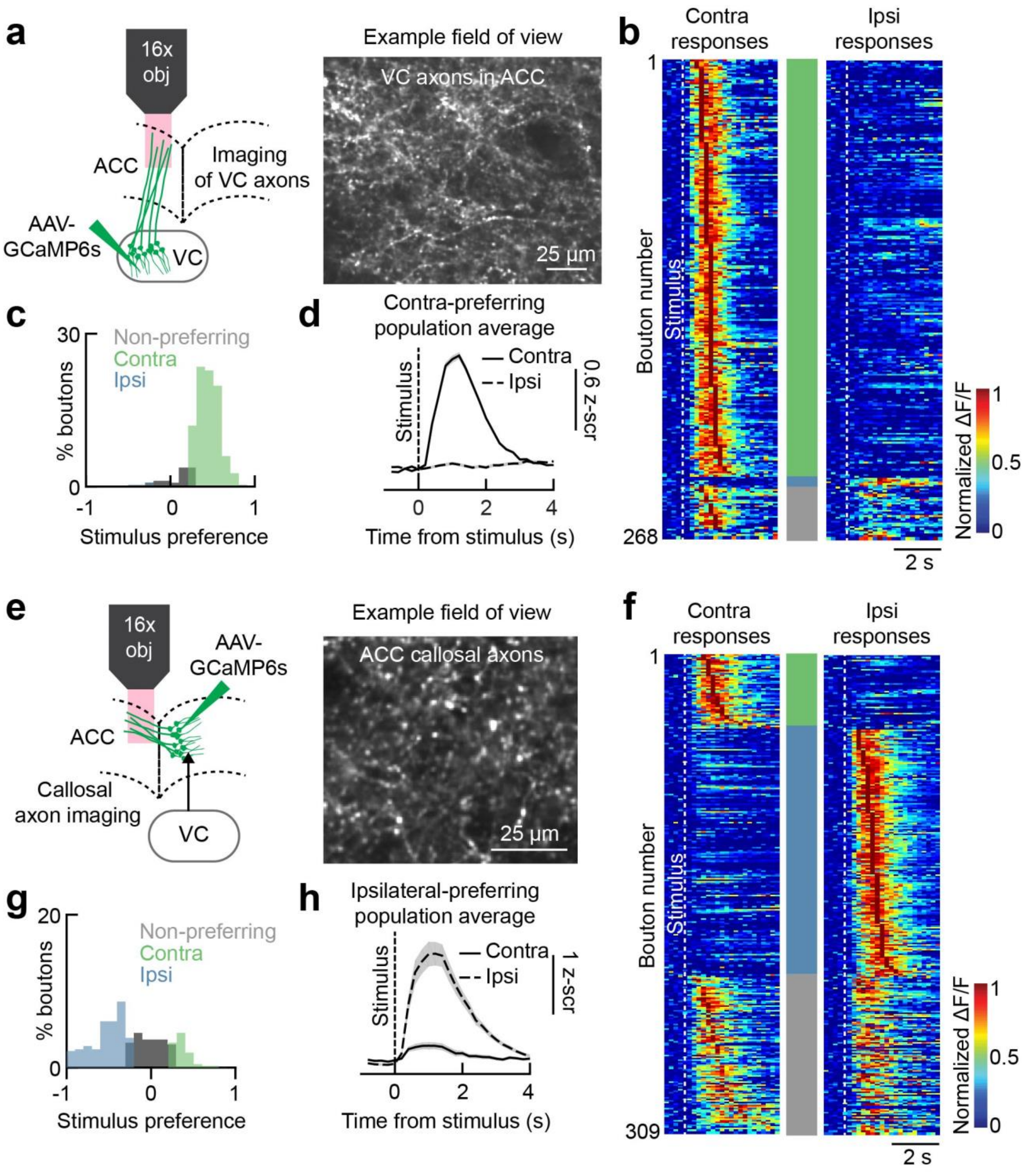

Supplementary Figure 1. Visually-evoked responses of VC and callosal inputs to the ACC. (**a**) Two- photon calcium imaging via a 16x objective (obj) of GCaMP6s-expressing visual cortex (VC) axons in the ACC. Similar labeling was observed in the 4 mice tested. (**b**) Trial-averaged responses of individual visually-responsive boutons to stimuli presented in hemifield contralateral

(contra) or ipsilateral (ipsi) to the recording site (black square, ~20º size, 1s). Responses are grouped by their stimulus preference (contra, green; ipsi, blue, non-preferring, gray) and sorted within each group by their peak response time. (**c**) AUROC analysis was used to calculate a stimulus preference score for each visually-responsive bouton. This score ranges from -1 (ipsi selective) to 1 (contra selective). Distribution of scores for 268 visually-responsive VC boutons in ACC from 4 mice is shown. (**d**) Population-averaged z-scored (z-scr) responses of contra-preferring boutons to contralateral (solid line) and ipsilateral (dashed line) stimuli (n = 233 boutons from 4 mice). Shading shows the standard error of the mean. (**e-h**) Same as **a-d**, except for callosal boutons. The representative labeling shown in e was similar to that observed in the 5 mice tested. In **g**, distribution of preference scores from 309 callosal boutons from 5 mice is shown; in **h**, n = 156 ipsi-preferring boutons from 5 mice. Shading shows standard error of the mean.

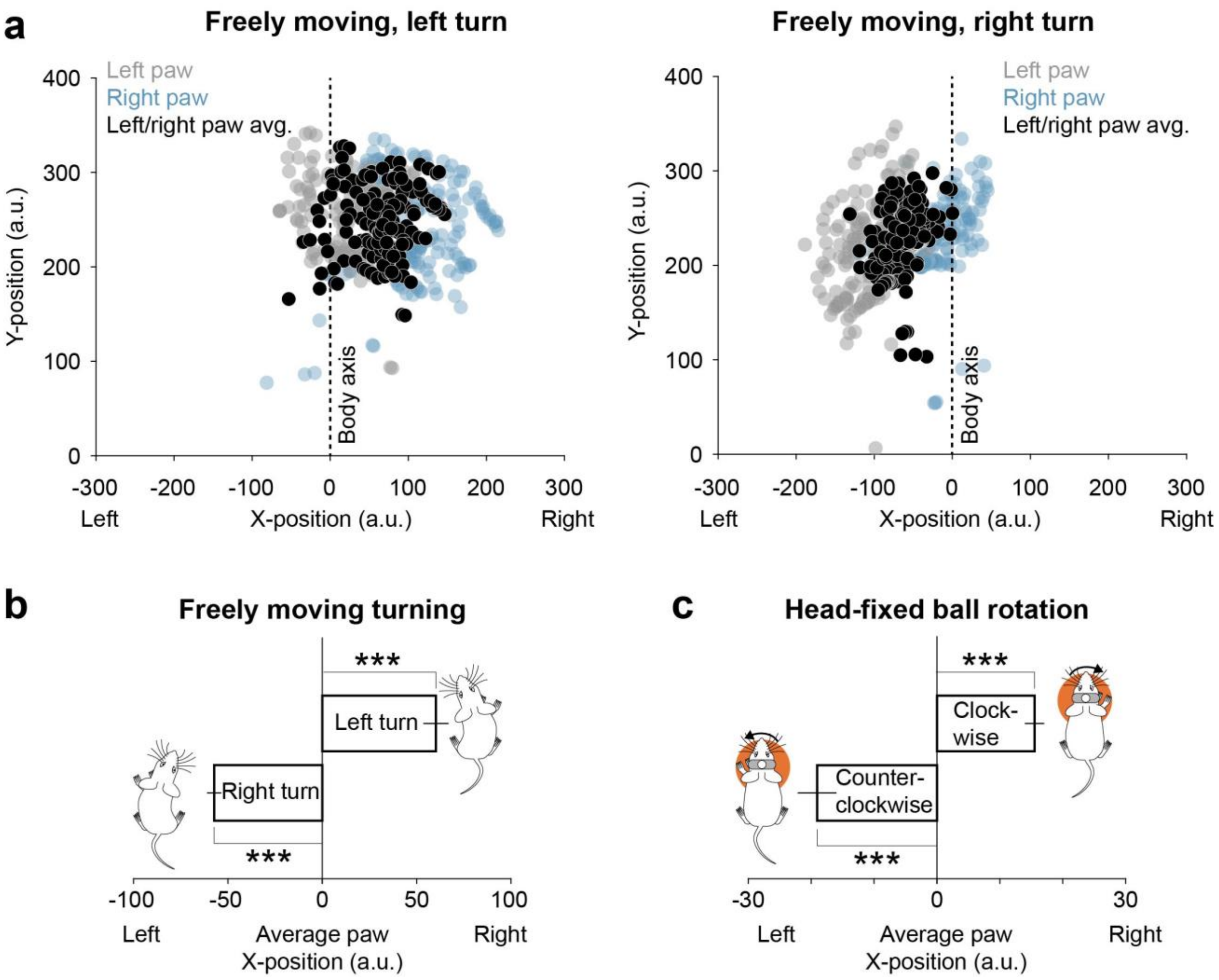

Supplementary Figure 2. Forepaw positions during freely moving turning and head-fixed ball rotations. (**a**) Individual dots represent left (gray) and right (blue) paw positions extracted from videos of freely moving mice during left and right turns. Each dot corresponds to the paw during individual frames of the videos. The average (avg) position of the left and right paws is shown with black dots. The dotted line shows the axis of the body, defined as a line drawn between the nose and the base of the tail (n = 14 turns for each direction from 3 mice). (**b**) The average X-position of the two paws is shown for right and left turns (n = 14 turns for each direction from 3 mice; left turn vs. zero, $p = 0.001$, $z = 3.30$; right turn vs. zero, $p = 0.001$, $z = -3.30$; left turn vs. right turn, $p = 0.001$, $z = 3.30$).***$p < 0.005$; two-tailed Wilcoxon signed-rank test against zero. (**c**) The same mice shown in A, B were video recorded during ball rotations while head-fixed. The average X-position of the two paws during counterclockwise and clockwise ball rotations are shown (n = 15 turns for each direction from 3 mice; clockwise vs. zero, $p = 0.0007$, $z = 3.41$; counterclockwise vs. zero, $p = 0.0007$, $z = -3.41$; CW vs. CCW, $p = 0.0007$, $z = 3.41$).***$p < 0.005$; two-tailed Wilcoxon signed-rank test against zero).

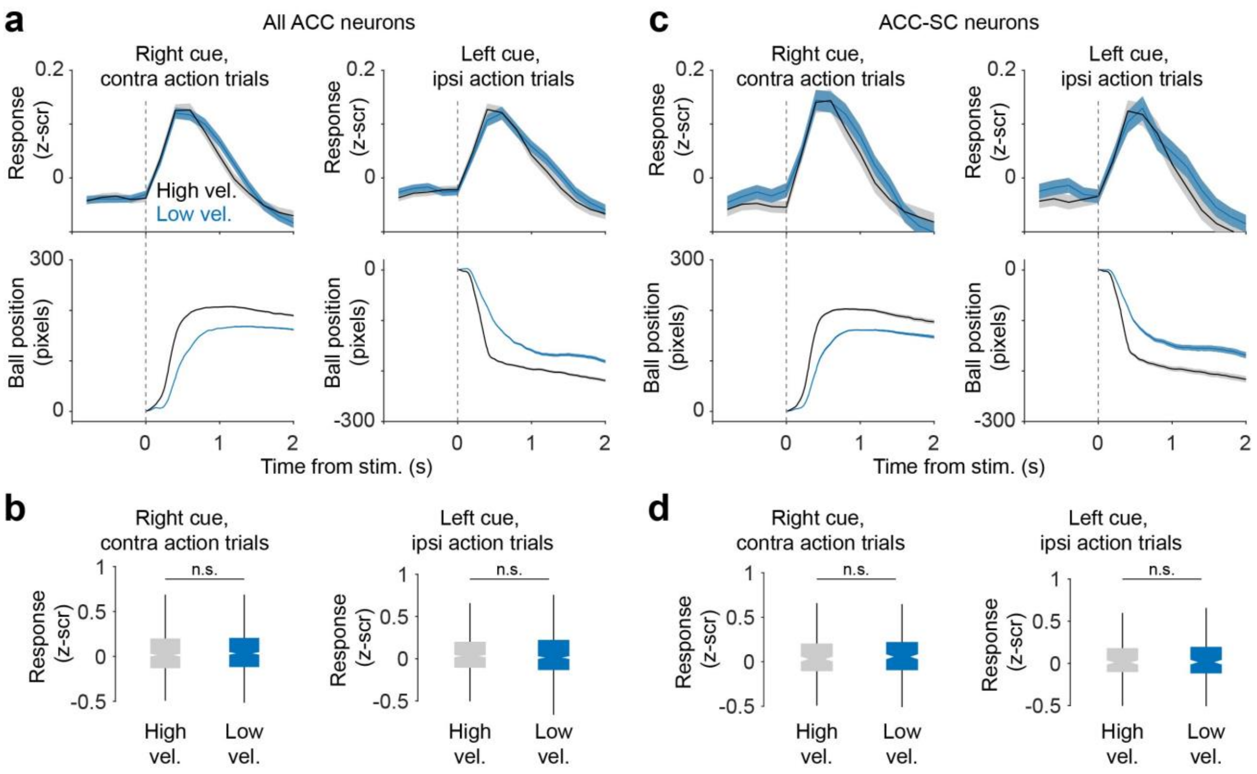

Supplementary Figure 3. Modulation of task responses by movement velocity. (**a**) Task responses and ball position trajectories on correct right cue and left cue trials are shown for high (black) and low (blue) velocity (vel) ball rotations (n = 1156 neurons from 5 mice). (**b**) z-scored (z-scr) DFF response was averaged over a 1s window after stimulus (stim) onset. Average responses for high and low velocity on right cue (p = 0.98, z = -0.027) and left cue (p = 0.41, z = 0.8176) trials is shown. Box plot elements: center line, median; box limits, upper and lower quartiles; whiskers, 1.5x interquartile range; outliers not shown. Data in **a** and **b** is from all ACC neurons (n = 1156 neurons from 5 mice). (**c, d**) Same as **a**, **b**, except for ACC-SC neurons (n = 303 neurons from 5 mice; right cue, p = 0.49, z = -0.69; left cue, p = 0.89, z = 0.14). Error bars or shading in all panels represent the standard error of the mean. Statistical testing with two-sided Wilcoxon signed-rank test.

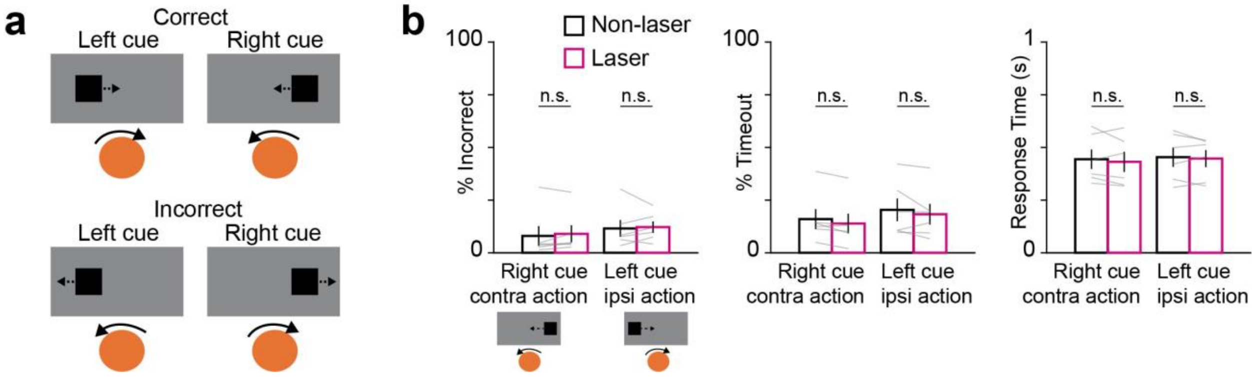

Supplementary Figure 4. Effect of light delivery on behavioral performance. (**a**) Schematic depicting correct and incorrect responses on the inward task. (**b**) Behavioral performance with light delivery (20mW) in the absence of opsins. Behavioral performance for non-laser and laser conditions on right cue and left cue trials. Incorrect performance (right cue, p = 0.201; left cue, p = 0.842), timeouts (right cue, p = 0.998; left cue, p = 0.078; n = 6 mice), and response time (right cue, p = 0.527; left cue, p = 0.647) are shown. Significance testing with permutation test. n.s., not significant. Significance evaluated at the Bonferroni-adjusted p-value of 0.025 with a two-tailed permutation test. Error bars represent the standard error of the mean.

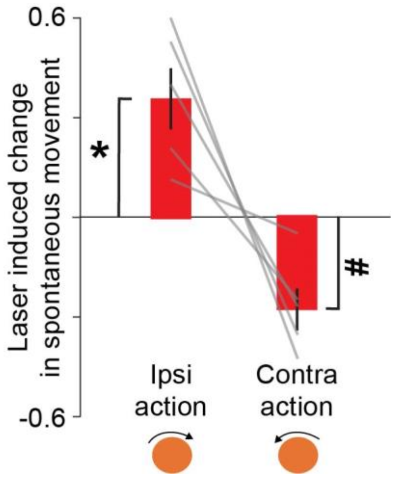

Supplementary Figure 5. SC inactivation during spontaneous movement. AAV5-Syn-Jaws was injected in the SC and a fiber optic was implanted over the injection site. Laser induced changes in ipsiversive (ipsi) and contraversive (contra) actions during spontaneous movements are shown (ipsiversive, *p = 0.045; contraversive, #p = 0.0517; n = 5 mice; two-tailed permutation test). Error bars are standard error of the mean.

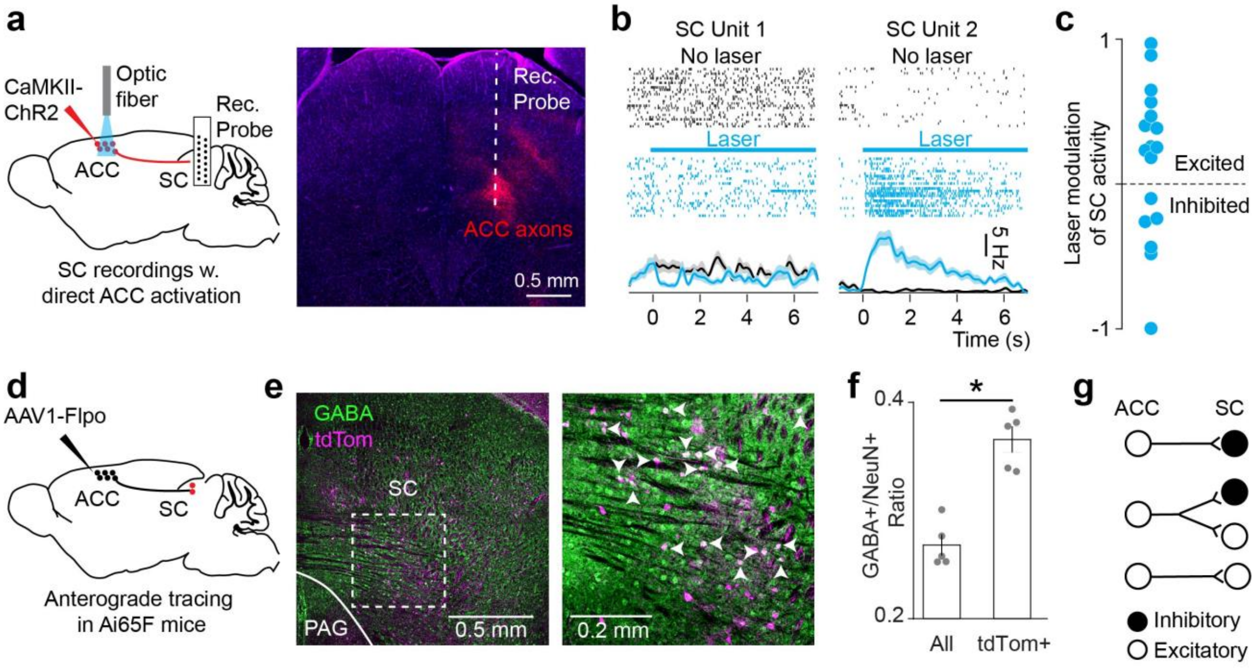

Supplementary Figure 6. Effect of ACC activation on SC activity. (**a**) AAV virus encoding CaMKII-ChR2-mCherry was injected unilaterally in the ACC and an optic fiber was implanted above the injection site. Extracellular recording probe was inserted in the superior colliculus (SC) on the same side as the ACC optic fiber. Similar expression and implant location was observed in the 3 mice tested. (**b**) Activity of two example SC units with and without photostimulation of the ACC. Shading shows the standard error of the mean across trials (**c**) Laser-modulation indices for SC units significantly modulated with ACC photoactivation. (**d**) SC neurons receiving inputs from the ACC were labeled by injecting AAV1-Flpo virus in the ACC of tdTomato reporter mice. (**e**) *Left*, SC neurons labeled with tdTomato (magenta). Imunnohistochemistry against GABA (green) shows inhibitory neurons. *Right*, higher magnification image of the area bounded by the dotted white square in left panel. Arrowheads mark neurons double-labeled with tdTomato and GABA. Similar labeling was observed in the 5 mice tested. (**f**) Proportion of all SC neurons (All) and tdTomato labeled (tdTom+) neurons (i.e., NeuN positive cells) that are co-labeled with GABA (*p = 0.0431, z = -2.023; n = 5 mice). Significance testing with two-tailed Wilcoxon signed-rank test. Error bars are standard error of the mean. (**g**) Proposed circuit schematic for ACC connectivity with excitatory and inhibitory SC neurons.

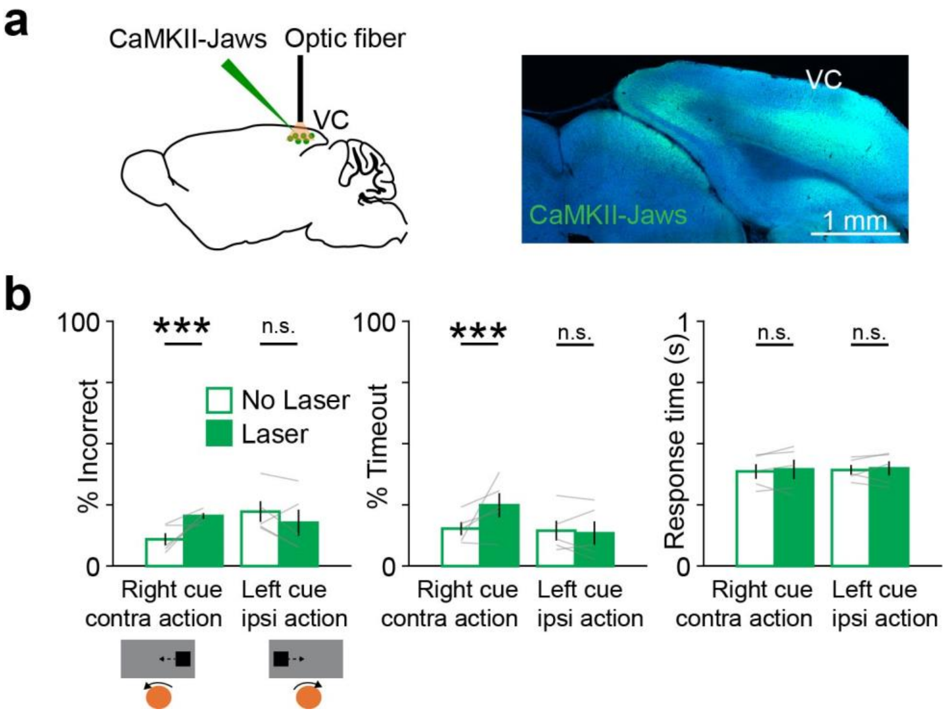

Supplementary Figure 7. Effect of VC inactivation on task performance. (**A**) AAV5-CaMKII-Jaws was injected in the visual cortex (VC), which was inactivated via yellow light through a chronic window. Similar labeling was observed in the 5 mice tested. (**B**) Behavioral performance for non-laser (unfilled) and laser (filled) conditions on right cue/contraversive action and left cue/ipsiversive action cue trials with inactivation of the VC (n = 5 mice). Incorrect performance (right cue, p = 0.001; left cue, p = 0.117), timeouts (right cue, p = 0.001; left cue, p = 0.496), and response time (right cue, p = 0.232; left cue, p = 0.966) are shown. Significance evaluated at Bonferroni adjusted p-value of 0.025 with a two-tailed permutation test. ***p < 0.005. Error bars are standard error of the mean.

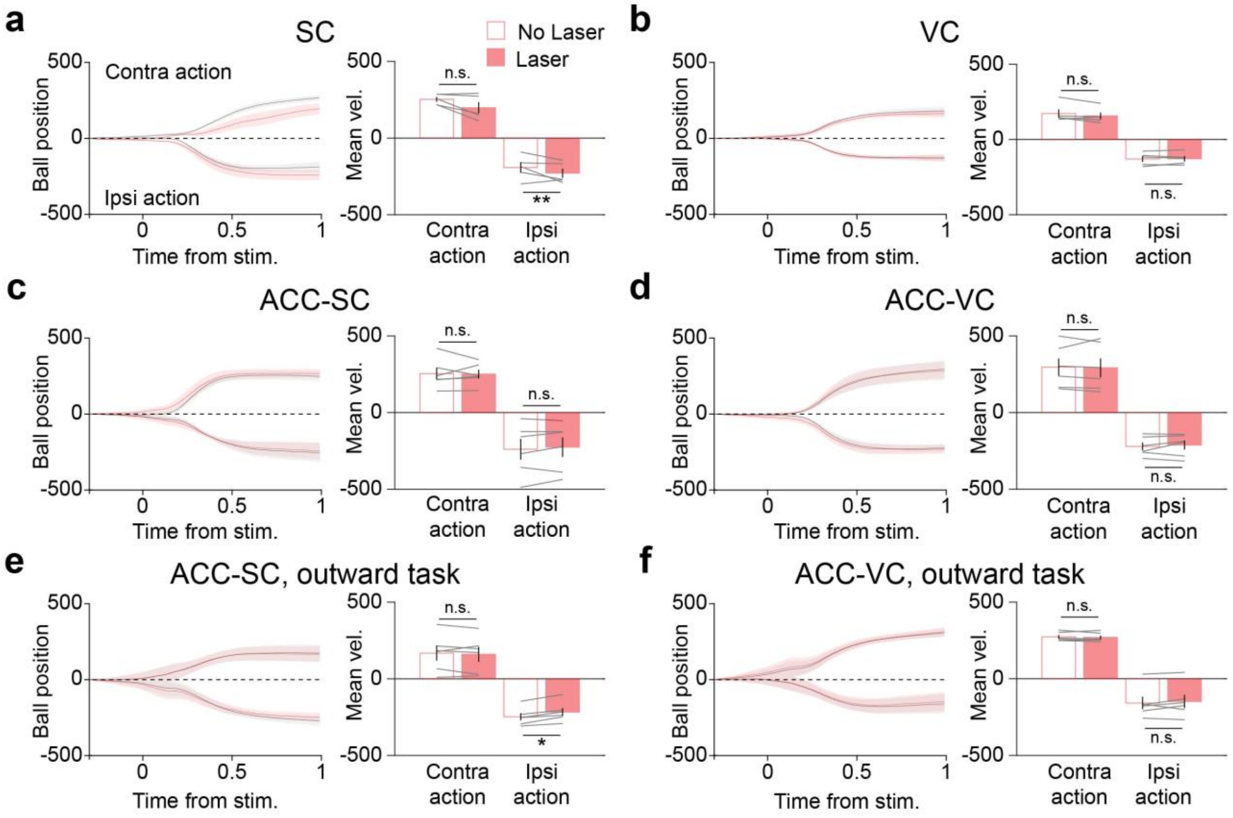

Supplementary Figure 8. Effect of optogenetic inactivations on ball movement trajectories. (**a**) *Left*, ball positions on contraversive (contra) and ipsiversive (ipsi) action trials with and without laser inactivation of the superior colliculus (SC). *Right*, mean instantaneous ball velocity (vel) from 0-1s after stimulus onset on the indicated trials with and without the laser (contra, $p = 0.24$; ipsi, $p = 0.006$; $n = 5$ mice). (**b-f**) Same as **b**, except for inactivation of the visual cortex (VC; contra, $p = 0.84$; ipsi, $p = 0.50$; $n = 5$ mice), ACC-SC (contra, $p = 0.60$; ipsi, $p = 0.13$; $n = 6$ mice), ACC-VC (contra, $p = 0.58$; ipsi, $p = 0.21$; $n = 6$ mice), ACC-SC outward task (contra, $p = 0.66$; ipsi, $p = 0.01$; $n = 6$ mice), and ACC-VC outward task (contra, $p = 0.23$; ipsi, $p = 0.74$; $n = 6$ mice). In all panels, significance was evaluated with Bonferroni-adjusted p-value of 0.025 with a two-tailed permutation test. Error bars and shading represent the standard error of the mean. *$p < 0.025$; **$p < 0.01$; n.s., not significant.
