# Supplementary material for: Distinct prefrontal top-down circuits differentially modulate sensorimotor behavior
Source: Nat Commun. 2020 Nov 26;11:6007. doi: 10.1038/s41467-020-19772-z (PMC7691329; doi:10.1038/s41467-020-19772-z)
Supplement: Supplementary file 3 — Reporting Summary [file 41467_2020_19772_MOESM3_ESM.pdf]

nature research

Corresponding author(s): Rafiq Huda, Mriganka Sur

Last updated by author(s): Sep 24, 2020

Reporting Summary

Nature Research wishes to improve the reproducibility of the work that we publish. This form provides structure for consistency and transparency in reporting. For further information on Nature Research policies, see our Editorial Policies and the Editorial Policy Checklist.

Statistics

For all statistical analyses, confirm that the following items are present in the figure legend, table legend, main text, or Methods section.

n/a	Confirmed	
☐	☒	The exact sample size (*n*) for each experimental group/condition, given as a discrete number and unit of measurement
☐	☒	A statement on whether measurements were taken from distinct samples or whether the same sample was measured repeatedly
☐	☒	The statistical test(s) used AND whether they are one- or two-sided<br>*Only common tests should be described solely by name; describe more complex techniques in the Methods section.*
☐	☒	A description of all covariates tested
☐	☒	A description of any assumptions or corrections, such as tests of normality and adjustment for multiple comparisons
☐	☒	A full description of the statistical parameters including central tendency (e.g. means) or other basic estimates (e.g. regression coefficient) AND variation (e.g. standard deviation) or associated estimates of uncertainty (e.g. confidence intervals)
☐	☒	For null hypothesis testing, the test statistic (e.g. F, t, r) with confidence intervals, effect sizes, degrees of freedom and P value noted<br>*Give P values as exact values whenever suitable.*
☒	☐	For Bayesian analysis, information on the choice of priors and Markov chain Monte Carlo settings
☒	☐	For hierarchical and complex designs, identification of the appropriate level for tests and full reporting of outcomes
☒	☐	Estimates of effect sizes (e.g. Cohen's d, Pearson's r), indicating how they were calculated

Our web collection on statistics for biologists contains articles on many of the points above.

Software and code

Policy information about availability of computer code

Data collection	Behavioral data were collected with custom scripts written in MATLAB (version 2015a) using Psychtoolbox-3.<br>Two-photon imaging was performed using the Prairie software (version 5.4).<br>Electrophysiology data were collected using the Plexon Recorder software (version 2.8.8).
Data analysis	Data analysis and statistical testing was done with custom scripts written in MATLAB (version 2017a).<br>Unit sorting was performed using MountainSort and Mountainview (version 3).<br>ImageJ (version 1.53b) was used for extracting fluorescence data.<br>ImageJ plugin Cell Counter was used for quantifying anatomical tracing experiments (version 2.2.2).<br>Forepaw tracking was done with DeepLabCut (version 2.1.8.2).<br>LIBSVM MATLAB library (version 3.22)

For manuscripts utilizing custom algorithms or software that are central to the research but not yet described in published literature, software must be made available to editors and reviewers. We strongly encourage code deposition in a community repository (e.g. GitHub). See the Nature Research guidelines for submitting code & software for further information.

Data

Policy information about availability of data

All manuscripts must include a data availability statement. This statement should provide the following information, where applicable:

- Accession codes, unique identifiers, or web links for publicly available datasets
- A list of figures that have associated raw data
- A description of any restrictions on data availability

Source data for Figs. 1c, 1g, 1i, 2c, 2g, 2h, 2i, 3d, 3e, 3f, 3g, 4b, 4d, 5a, 5c, 5d, 6b, 6c, 6d, 6e and Supplementary Figs. 1d, 1h, 2a, 2b, 2c, 3b, 3d, 4, 5, 6c, 6f, 7b, 8a, 8b, 8c, 8d, 8e, and 8f are included in the Source Data file. The data that support the findings of this study are available from the corresponding author upon reasonable request.

Field-specific reporting

Please select the one below that is the best fit for your research. If you are not sure, read the appropriate sections before making your selection.

[x] Life sciences [ ] Behavioural & social sciences [ ] Ecological, evolutionary & environmental sciences

For a reference copy of the document with all sections, see nature.com/documents/nr-reporting-summary-flat.pdf

Life sciences study design

All studies must disclose on these points even when the disclosure is negative.

Sample size	Sample sizes were not predetermined. Our previous work (Goard et al., 2016; Pho et al., 2018) with two-photon calcium imaging and optogenetic manipulations suggests that the sample sizes used here are sufficient to detect the role of different circuits in visuomotor behavior.
Data exclusions	Analysis of ACC task responses excluded behavioral sessions with <5 error trials. This was required to compare task responses on trials with opposing actions but the same cue. This exclusion criteria was not pre-established since we could not control how many error trials mice would do.<br>Optogenetic sessions were excluded if behavioral performance on non-laser trials was below 60% correct. This was to ensure the behavioral deficits were due to optogenetic manipulations and not poor baseline performance. This exclusion criteria was not pre-established.
Replication	All experiments were repeated in at least two subjects. All attempts at replication were successful.
Randomization	Mice with appropriate genotypes were randomly assigned to different experimental groups (i.e., optogenetic inactivation of specific brain areas/projections). Stimuli were delivered randomly during the task. Subset of trials were randomly selected for photostimulation.
Blinding	Data collection and analysis were not performed in a blinded fashion. Blinding was not feasible in this study since the experimenter had to inject viruses/put implants in specific brain structures and then subsequently target them with light for photoinhibition and use knowledge about identity of the laser trials to perform the analysis.

Reporting for specific materials, systems and methods

We require information from authors about some types of materials, experimental systems and methods used in many studies. Here, indicate whether each material, system or method listed is relevant to your study. If you are not sure if a list item applies to your research, read the appropriate section before selecting a response.

Materials & experimental systems

n/a	Involved in the study	
[ ]	[x]	Antibodies
[ ]	[x]	Eukaryotic cell lines
[x]	[ ]	Palaeontology and archaeology
[ ]	[x]	Animals and other organisms
[x]	[ ]	Human research participants
[x]	[ ]	Clinical data
[x]	[ ]	Dual use research of concern

Methods

n/a	Involved in the study	
[x]	[ ]	ChIP-seq
[x]	[ ]	Flow cytometry
[x]	[ ]	MRI-based neuroimaging

Antibodies

Antibodies used	Primary GABA antibody (rabbit anti-GABA, 1:500, polyclonal, Sigma: catalog # A2052, lot # 078M4767V)<br>Primary NeuN antibody (guinea pig anti-NeuN, 1:500, polyclonal, Synaptic Systems: catalog # 266-004, lot # 2-18)<br>Secondary antibody (goat anti-rabbit IgG AlexaFluor 488, 1:500, Invitrogen: catalog # A11034, lot # 1812166)

	Secondary antibody (goat anti-guinea pig IgG AlexaFluor 647, 1:500, Invitrogen: catalog # A21450, lot # 1841758)
Validation	Rabbit anti-GABA has been validated by the manufacturer ("Expression of GABA in neucortical cells harvested from the brains of E19 day old rat embyros was detected by immunofluorescence using rabbit anti-GABA antibody. Triple IF staining was performed with the anti-GABA antibody and two anti-GAD antibodies. Expression of GABA was analyzed in cells isolated from the pallium of various animals including rats, mice, rabbits, guinea pigs, and lizards by immunohistochemistry") and previous literature: Adhikari, Avishek et al. “Basomedial amygdala mediates top-down control of anxiety and fear.” Nature vol. 527,7577 (2015): 179-85. doi:10.1038/nature15698<br><br>Guinea pig anti-NeuN has been validated in previous literature: Pao, Ping-Chieh et al. “HDAC1 modulates OGG1-initiated oxidative DNA damage repair in the aging brain and Alzheimer's disease.” Nature communications vol. 11,1 2484. 18 May. 2020, doi:10.1038/s41467-020-16361-y<br><br>Goat anti-rabbit IgG AlexaFluor 488 has been validated in previous literature: Duan, Gui-Fang et al. “Signal peptide represses GluK1 surface and synaptic trafficking through binding to amino-terminal domain.” Nature communications vol. 9,1 4879. 19 Nov. 2018, doi:10.1038/s41467-018-07403-7<br><br>Goat anti-guinea pig IgG AlexaFluor 647 has been validated in previous literature: Smith, Harvey W et al. “An ErbB2/c-Src axis links bioenergetics with PRC2 translation to drive epigenetic reprogramming and mammary tumorigenesis.” Nature communications vol. 10,1 2901. 1 Jul. 2019, doi:10.1038/s41467-019-10681-4

Eukaryotic cell lines

Policy information about cell lines

Cell line source(s)	HEK 293T cells were from ATCC (CRL-11268).
Authentication	Cell line was authenticated by ATCC.
Mycoplasma contamination	Cells were not tested for mycoplasma contamination.
Commonly misidentified lines (See ICLAC register)	No commonly misidentified lines were used.

Animals and other organisms

Policy information about studies involving animals; ARRIVE guidelines recommended for reporting animal research

Laboratory animals	The following mice were used in this study: wildtype (C57BL/6J), Jackson # 000664; Ai94D (B6.Cg-Igs7tm94.1(tetO-GCaMP6s)/J), Jackson # 024104; Tg(Rbp4-cre)KL100Gsat/Mmucd, MMRC # 031125-UCD; Ai65(RCFL-tdT)-D, Jackson # 021875; B6.129S4-Meox2tm1(cre)Sor/J, Jackson # 003755. Mice of either sex at age 2-6 months at the time behavioral training started were used.
Wild animals	Wild animals were not used in this study.
Field-collected samples	Field-collected samples were not used in this study.
Ethics oversight	All experimental procedures performed on mice were approved by the Massachusetts Institute of Technology Animal Care and Use Committee.

Note that full information on the approval of the study protocol must also be provided in the manuscript.
